# Interplay between Phosphatases and the Anaphase-Promoting Complex/Cyclosome in Mitosis

**DOI:** 10.3390/cells8080814

**Published:** 2019-08-02

**Authors:** Meghna Kataria, Hiroyuki Yamano

**Affiliations:** UCL Cancer Institute, University College London, London WC1E 6DD, UK

**Keywords:** phosphatase, kinase, anaphase-promoting complex/cyclosome (APC/C), cyclin-dependent kinase 1 (Cdk1), cyclin, mitosis, cell cycle, phosphorylation, ubiquitylation

## Abstract

Accurate division of cells into two daughters is a process that is vital to propagation of life. Protein phosphorylation and selective degradation have emerged as two important mechanisms safeguarding the delicate choreography of mitosis. Protein phosphatases catalyze dephosphorylation of thousands of sites on proteins, steering the cells through establishment of the mitotic phase and exit from it. A large E3 ubiquitin ligase, the anaphase-promoting complex/cyclosome (APC/C) becomes active during latter stages of mitosis through G1 and marks hundreds of proteins for destruction. Recent studies have revealed the complex interregulation between these two classes of enzymes. In this review, we highlight the direct and indirect mechanisms by which phosphatases and the APC/C mutually influence each other to ensure accurate spatiotemporal and orderly progression through mitosis, with a particular focus on recent insights and conceptual advances.

## 1. Introduction

From the outset, the process of cell division might seem mired in extraordinary complexity. Following a series of distinct steps, a eukaryotic cell is able to duplicate its genetic content and partition it among its two daughters along with other organelles with remarkable precision. Decades of research has sought to understand the molecular and physico-chemical underpinnings of the ‘cell division cycle’: from DNA replication, transitory structures such as the mitotic spindle devoted to its distribution, to control systems that monitor the fidelity of these processes. The realization that reversible protein modifications such as phosphorylation and ubiquitylation are at the heart of the cell cycle engine has demystified many of its aspects.

The primary focus of this review is to highlight the roles of two classes of protein modifying enzymes that are central to accurate mitosis and the complex interplay between them: protein phosphatases and the E3 ubiquitin ligase, anaphase-promoting complex/cyclosome (APC/C), with a focus on recent advances. These two modifiers act in concert with numerous mitotic kinases to orchestrate various events of mitosis.

### 1.1. Protein Phosphatases—Ubiquitous Enzymes that Regulate Cell Physiology and Division

The discovery of phosphatases, then called ‘prosthetic group’-removing or PR enzymes, predated that of kinases [1]. Abundant in rabbit muscle and spleen, the phosphatase contemporaneously referred to as PP1 was shown to remove an inorganic ‘prosthetic group’ from muscle glycogen phosphorylase, converting it to a less active form. A decade later, inorganic phosphate was hinted at [2] and then proven [3] as the identity of the removable prosthetic group. It is now clear that hydroxyl-containing amino acids, i.e., Ser, Thr and Tyr, undergo phosphorylation by protein kinases, while phosphatases catalyze phosphate removal, thereby controlling steady-state levels of this modification.

**Figure 1:**
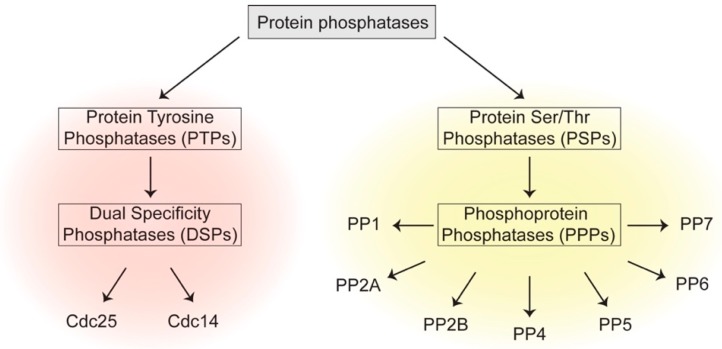
Protein phosphatases involved in mitosis. Protein phosphatases can be divided into two superfamilies: Protein Tyrosine Phosphatases (PTPs) and Protein Ser/Thr Phosphatases (PSPs). Although these families contain a vast array of phosphatases, those pertinent to mitosis and discussed herein are shown.

The years following the discovery of PP1 witnessed the identification of many different classes of phosphatases which can be broadly divided into two groups: one is Protein Tyrosine Phosphatases (PTPs), which comprises classical PTPs and Dual Specificity Phosphatases (DSP) that catalyze dephosphorylation of p-Tyr, or p-Tyr, p-Ser and p-Thr residues, respectively [4]. The other family is Protein Serine/Threonine Phosphatases (PSPs) that dephosphorylate p-Ser and p-Thr residues [5] (Figure 1). The human genome contains 189 protein phosphatases, encompassing 10 different protein structural folds and a variety of catalytic mechanisms [6]. This is in contrast to 518 identified human kinases, whose catalytic domains trace back to a single structural fold [7].

**Figure 2:**
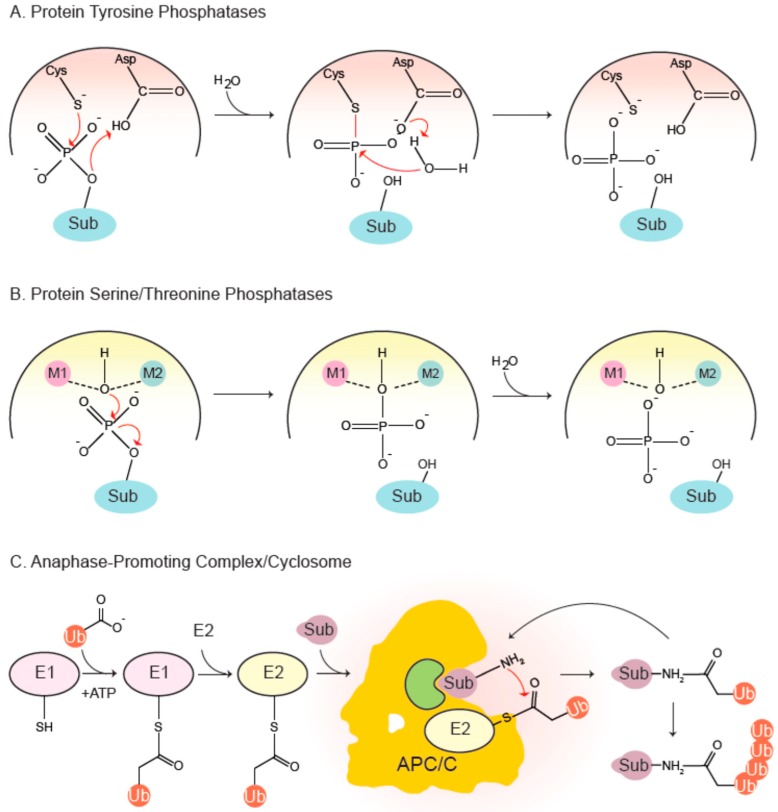
Catalytic mechanisms of protein tyrosine phosphatase (PTP), protein serine/threonine phosphatase (PSP) and anaphase-promoting complex/cyclosome (APC/C). (**A**) PTP; the sulphur atom of the catalytic cysteine, part of the signature PTP loop sequence, initiates a nucleophilic attack on the phosphoryl group of the substrate and forms a phosphoryl-enzyme intermediate, while the dephosphorylated substrate receives a proton from the aspartate residue that forms part of the WPD loop. A water molecule leads to hydrolysis of the phosphoryl group. (**B**) PSP; enzyme-bound two metal ions (e.g., Fe^2+^, Zn^2+^ or Mn^2+^) coordinate a water molecule that mounts a nucleophilic attack on the substrate phosphoryl group, leading to substrate dephosphorylation. (**C**) APC/C E3 ubiquitin ligase; ubiquitylation requires the action of three enzymes (E1, E2 and E3). First, the E1 ubiquitin-activating enzyme uses ATP and catalyzes ubiquitin C-terminal adenylation. Then, an active site cysteine on the E1 enzyme attacks the C-terminus of ubiquitin and forms a thioester-linked E1~ubiquitin intermediate. The activated ubiquitin is then transferred to an active site cysteine of an E2 ubiquitin-conjugating enzyme. Finally, co-activator bound-APC/C (E3 ubiquitin ligase) simultaneously binds the ubiquitin-charged E2 and a substrate, leading to transfer of the ubiquitin molecule to a primary amine within the substrate. This process repeats several times, yielding polyubiquitylated substrate. Abbreviations: Sub, substrate.

The PTP family is a canopy encompassing diverse phosphatases all of which share the signature motif HCx_5_R (PTP loop). The sulfur atom of the catalytic cysteine initiates a nucleophilic attack on the phosphorylated substrate (Figure 2), forming a thiophosphate intermediate that is experimentally observable. A conserved aspartate, part of the WPD loop, donates a proton to the dephosphorylated protein, leading to its stabilization and eventual dissociation. Next, a water molecule hydrolyzes the thiophosphate-enzyme intermediate, again mediated by the aspartate, that accepts a proton from the water molecule [4].

The PSP family includes a phosphoprotein phosphatase (PPP) subfamily which comprises of PP1, PP2A, PP2B/calcineurin, PP4, PP5, PP6 and PP7. These phosphatases have been implicated in diverse functions in the cell cycle and beyond. Their catalytic sites adopt similar folds and essentially follow the same mechanism of catalysis (Figure 2). Two divalent metal ions—Fe^2+^ and Zn^2+^ in native enzymes, and two Mn^2+^ ions in recombinant ones—are coordinated by a number of conserved residues involved in metal binding [8,9]. The metal-bound catalytic center holds two water molecules, one of which is activated to mount a nucleophilic attack on the phosphate group of the substrate, thereby leading to its dephosphorylation in a single step [10]. PP1 and PP2A are the most-studied PPPs controlling the cell cycle. PP1 contains surface grooves radiating out from the metal-coordinating center that bind a plethora of degenerate short docking motifs such as RVxF and SILK, to name a few [11]. They mediate interaction of PP1 with hundreds of partner proteins, many of which typically employ multiple docking sites to associate with the phosphatase and conscript it for various functions. Indeed, it has recently been reported that PP1 starts life as a trimer with inhibitor-3 and Sds22 acting as inhibitory chaperones, which are then exchanged for other regulatory proteins [12,13,14]. A restricted set of components constitute the trimeric PP2A holoenzyme: a scaffold A subunit, a catalytic C subunit and a choice of four families of regulatory B subunits, each with multiple isoforms. B55 and B56 families are important in cell division.

The examples of PP1 and PP2A highlight a leitmotif in phosphatase function, including ones controlling cell division: in vivo they tend to function as obligate multimeric enzymes. The catalytic subunit typically associates with one or more additional subunit. These additional subunits can act as scaffolds, regulators or inhibitors, thereby fundamentally altering the properties of the phosphatase. Since early experiments were often performed with the catalytic subunit, phosphatases appeared to show little discrimination towards substrates. This observation, coupled with the relatively few numbers of known phosphatases in comparison to kinases, led to the erroneous yet resilient view that phosphatases are promiscuous housekeeping enzymes working in the background as their kinase counterparts orchestrate major cellular events. In theory, this could indeed serve as an organizational principle in cellular control. For instance, it is well established that oscillating activity of cyclins in complex with cyclin-dependent kinases (Cdks) propels the cell through various transitions by orderly phosphorylation of substrate proteins [15,16], with the tacit assumption that many of these modifications are reversed by background phosphatase activity. Recent research, however, has challenged this view, and now places these enzymes as sophisticated molecular participants. Substrate specificities of phosphatases, imbued by both the active site and docking motif receptors within associated regulatory proteins, are intimately linked to their function [17,18,19,20,21,22,23,24,25,26,27].

Thousands of unique phosphorylation events on almost three-quarters of the proteome have been reported, regulating division and other cellular functions [19,28,29].

### 1.2. The Multiprotein Complex APC/C is a Master Regulator of the Cell Cycle

In addition to selective phosphorylation, selective degradation of proteins provides a major avenue for control of the cell cycle. A few decades following the discovery of phosphatases, it was found that cells selectively target proteins for degradation by marking them with covalently-attached chains of ubiquitin, a small, heat-stable protein [30,31]. These chains are recognized and irreversibly destroyed by the 26S proteasome. Degradation of mitotic cyclins and Cdk1 inactivation during mitotic exit was then attributed to the ubiquitin-protease system [32], thus marrying together the fields of protein phosphorylation and ubiquitylation in cell cycle control. In fact, ubiquitin-mediated degradation was found to be the impetus for cytological anaphase [33], triggered by destruction of securin [34,35], an inhibitory chaperone for the protease separase. Once released, separase cleaves the kleisin subunit of cohesin rings that hold sister chromatids together [36].

The attachment of ubiquitin, ‘ubiquitylation’, is a multi-step process with sequential actions of three enzymes (E1-E2-E3) [37,38,39]: The C-terminal glycine residue of ubiquitin is first activated by an E1 ubiquitin-activating enzyme in an ATP-dependent process and then covalently attached to the active cysteine residue of the E1 by formation of a thioester-ubiquitin conjugate. The activated ubiquitin is then transferred to the active site cysteine of an E2 ubiquitin-conjugating enzyme. Finally, an E3 ubiquitin ligase coordinates the transfer of ubiquitin to selected substrates by simultaneously binding a ubiquitin-charged E2 and a substrate in a productive manner (Figure 2). Attachment of ubiquitin requires a primary amine within the substrate and typically occurs on lysine residues or the N-terminal initiator methionine. The C-terminal glycine of ubiquitin forms an isopeptide bond with an ε-amine of the substrate. Further ubiquitylation can occur on either the N-terminus or any of the seven lysine residues present within the attached ubiquitin molecule. Polyubiquitylation leads to formation of different types of ubiquitin chains, leading the protein down different trajectories, one of which is irreversible degradation [39].

Deubiquitylating enzymes (DUBs) disassemble ubiquitin chains on substrates, providing another avenue for cellular control and homeostasis. There are about 93 different DUBs in the human genome, compared to about 600 E3 ubiquitin ligases [40,41], drawing parallels with reversible phosphorylation in that there is a discrepancy in numbers of enzymes that add a modification compared to ones that remove it.

The anaphase-promoting complex/cyclosome (APC/C) was found to be the E3 ubiquitin ligase responsible for degradation of Cyclin B during mitosis [42,43,44] by which the master regulatory kinase of mitosis, Cdk1-Cyclin B is switched off, driving cells out of mitosis. The APC/C is a gigantic 1.22 MDa multi-subunit enzyme belonging to the Cullin-RING family of E3 ligases. In vertebrates, it contains 14 highly conserved subunits—Apc1-8, Apc10-13, Apc15 and Apc16—five of which are present as dimers. Apc7 is absent from fission and budding yeast complexes. The APC/C itself is not catalytically active as a ubiquitin ligase. It becomes active only when it associates with a ‘co-activator’ protein belonging to the Cdc20/Fizzy family, such as Cdc20 or Cdh1. It should be noted that co-activators are not stoichiometric components of the APC/C, but their association with the APC/C is essential for its E3 ligase function [45,46,47]. Several organisms also contain meiosis-specific co-activators, such as Ama1 in budding yeast [48], Mfr1/Fzr1, Fzr2 and Fzr3 in fission yeast [49,50], Cort in *Drosophila* [51], etc. Co-activators not only act as receptors for the substrate protein to be modified, but also act as catalytic activators and induce structural rearrangements essential for E2 binding and ubiquitin transfer [52,53]. Some of the APC/C subunits had already been identified budding yeast genetic screens conducted by Hartwell et al. for temperature sensitive mutations that cause cell cycle arrest at elevated temperatures [54]. Perhaps unsurprisingly, this approach also identified the essential budding yeast cell cycle phosphatase Cdc14.

Recent structural studies have illuminated the overall architecture and subunit arrangement of the APC/C holocomplex. It adopts a hollow triangular shape, with the co-activator binding into this nook. Together with the core subunit Apc10, a co-activator forms the substrate recognition module of the enzyme and binds short linear degradation signals, or ‘degrons’ such as the destruction box (D-box) or the KEN-box present within intrinsically unstructured regions of target proteins [32,55,56,57,58]. The catalytic core module is composed of the Cullin-RING subunits Apc2-Apc11, respectively. The rest of the complex is devoted to co-activator recruitment and scaffolding functions, which are thought to coordinate optimal placement of the co-activator, the catalytic module and E2 enzymes for productive catalysis. The tetratricopeptide (TPR) lobe (the substrate recognition sub-complex) consists of the TPR repeat-containing proteins Apc3 (Cdc27), Apc6 (Cdc16), Apc7 (situated at the apex of the APC/C triangle; absent in yeast) and Apc8 (Cdc23) proteins that form homodimers, acting as a scaffold for co-activator binding. On the other hand, the multi-domain subunit Apc1, in conjunction with Apc4 and Apc5, forms a platform that bridges the catalytic module Apc2-Apc11 and the TPR lobe in a catalytically favorable conformation. This geometry also enables cell cycle-controlled binding of the APC/C inhibitors: the Mitotic Checkpoint Complex (MCC), the main effector of a surveillance mechanism called the Spindle Assembly Checkpoint (SAC) and Emi1 (early mitotic inhibitor), a metazoan APC/C inhibitor which is a vertebrate homologue of Rca1 (regulator of cyclin A) [59,60,61,62,63,64].

**Figure 3:**
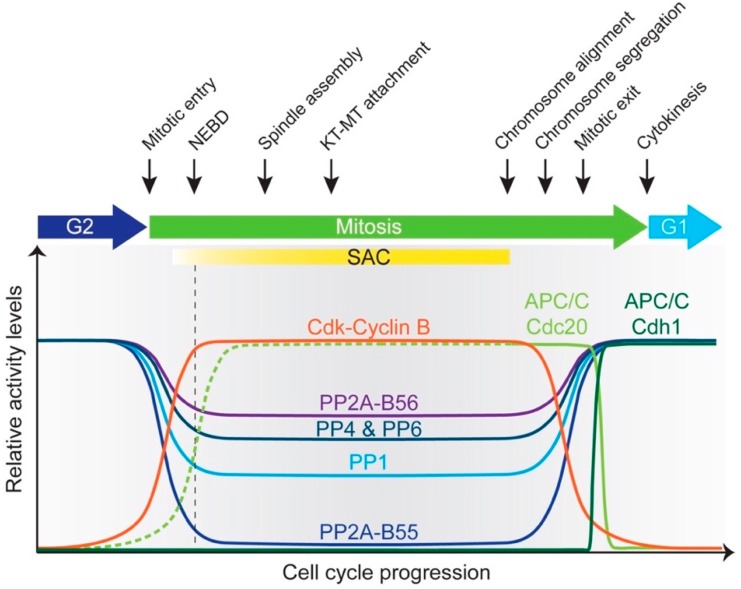
Activity dynamics of cell cycle phosphatases and the APC/C throughout mitosis. Only major serine/threonine phosphatases are depicted. Precise activity dynamics of PP2A-B56, PP4 and PP6 are not well understood in the context of other cell cycle events. During early mitosis, the APC/C-Cdc20 activity (dotted light green line) is held in check by the spindle assembly checkpoint (SAC; yellow bar) until correct bipolar attachment of chromosomes to the mitotic spindle is achieved. Only substrates that can directly bind the APC/C (such as Cyclin A and Nek2A) are degraded at this stage. Once the SAC is silenced, the APC/C-Cdc20 becomes fully active (solid light green line) and ubiquitylates substrates such as securin and Cyclin B for degradation. Most cell cycle phosphatases are under regulatory control of Cdk1, albeit with different mechanisms, in order to avoid futile phosphorylation events in mitosis. See text for details. Abbreviations: KT-MT, kinetochore-microtubule; NEBD: nuclear envelope breakdown.

In humans, it has been reported that 170 proteins are ubiquitylated by the APC/C, likely to be an underestimate [65]. Due to its importance in regulating entry into and exit from mitosis, cytokinesis and G1/S transition, APC/C activity is strictly controlled during the cell cycle. During G1, APC/C-Cdh1 activity not only prevents premature entry into S-phase by degrading Cyclins and Skp2, but also regulates replication origin assembly by degrading regulators of DNA replication (e.g., geminin). At G1/S, multiple mechanisms, such as direct Cdh1 phosphorylation, Emi1 inhibitor binding and Cdh1 degradation, collaborate to shut off APC/C-Cdh1 activity and trigger S-phase. Emi1 continues to inhibit APC/C-Cdh1 throughout G2. In mitosis, prior to metaphase, APC/C-Cdc20 activity is restrained by MCC binding until all of the chromosomes are bi-oriented at the metaphase plate. While SAC prevents degradation of most substrates, Cyclin A and Nek2A bypass it by directly binding the APC/C [66,67]. Once the SAC is satisfied, APC/C-Cdc20 is unleashed from inhibition to degrade critical substrates such as Cyclin B and securin, heralding the onset of anaphase [68,69,70,71]. The latter stages of mitotic exit witness Cdh1 activation due to its dephosphorylation by reactivated phosphatases. Cdh1 replaces Cdc20 as the co-activator for the remainder of mitosis and targets the latter for destruction (Figure 3).

Unidirectional and orderly transitions through cell cycle phases are critical to our initial question of how cells divide in an error-free manner. Concerning mitotic exit, studies in both yeast and vertebrates show that APC/C-mediated proteolysis and dephosphorylation work in concert to ensure irreversible transition through this phase and establish a biochemically G1 state [72,73].

The APC/C and phosphatases exhibit considerable crosstalk during mitosis; they influence each other directly and indirectly. Broadly, APC/C activation during mitosis requires phosphorylation and is opposed by phosphatase action. Association of co-activators with the APC/C, however, requires them to be dephosphorylated by phosphatases. Phosphatases are also essential to silence the SAC and further promote APC/C activation. Active APC/C catalyzes mitotic cyclin degradation (inactivation of Cdk1) which then leads to phosphatase reactivation. These observations outline the interlinked nature of an extensive network of phosphorylation and ubiquitylation throughout M-phase and will be explored in greater detail in the following sections. Readers interested in detailed kinase and phosphatase crosstalk governing kinetochore-microtubule attachments and the SAC that are beyond the scope of this review are pointed to recent excellent comprehensive reviews [74,75,76,77,78].

## 2. G2/M Transition

### 2.1. Cdc25 Promotes Cdk1-Cyclin B Activation and Mitotic Entry

As mentioned previously, a quantitative increase in Cdk activity from G1 to M-phase triggers sequential passage through the cell cycle. Transition from G2 to M-phase requires an explosive, switch-like activation of Cdk1-Cyclin B. During G2, although the synthesis of Cyclin B increases due to transcriptional activation, concomitant increase in associated kinase activity requires removal of two inhibitory phosphates on the Cdk1 subunit: p-Thr14 and p-Tyr15. Phosphorylation of these residues prevents catalysis by causing ATP misalignment [79], and is performed by the inhibitory kinases Wee1 and Myt1. The dual-specificity PTP Cdc25 dephosphorylates these residues. Cdk1-Cyclin B catalyzes its own activation by phosphorylating both Wee1 and Cdc25 to opposite effects: inactivating the former but activating the latter, thus generating double negative and positive feedback loops. In addition, PP2A has long been known as a negative regulator of Cdk1 activation [80,81,82,83]. These activities underlie a bistable switch leading to swift Cdk1 activation and irreversible mitotic commitment [84,85], although studies in *Xenopus* indicate that preceding Cdk1-Cyclin A-mediated phosphorylation sets in motion mitotic commitment [86].

In humans, three Cdc25 paralogues exist; Cdc25A, Cdc25B and Cdc25C and perform distinct but overlapping functions in Cdk activation. Active Cdk1-Cyclin B first emerges at centrosomes, catalyzed by Cdc25B. In fact, centrosome splitting proceeds a few minutes after emergence of active kinase in unperturbed cells [87]. In a spatial positive feedback loop, partially activated cytoplasmic pool of Cdk1-Cyclin B then translocates into the nucleus driven by phosphorylation of the Cyclin N-terminus, where it eventually promotes nuclear envelope breakdown [88,89,90]. Nuclear pools of Cdc25A and Cdc25C promote further activation of the kinase.

The activity and subcellular localization of these phosphatases are themselves modified through phosphoregulation and proteolysis. For instance, phosphorylation of Cdc25C by Cdk1 and Polo-like kinase (Plk) increases its activity. Additionally, Cdc25C phosphorylation by a number of kinases at a 14-3-3 consensus sequence leads to its cytoplasmic retention and catalytic inactivation by these adaptor proteins [91,92]. An initial Cdk1-mediated phosphorylation allows PP1 binding to Cdc25C via its N-terminal RVxF motif. In a phosphatase relay, PP1 dephosphorylates the 14-3-3 docking site within the protein, thereby relieving Cdc25C from inhibition to dephosphorylate Cdk1 [93,94]. At the end of mitosis, Cdc25A is degraded by APC/C-Cdh1, thus further promoting Cdk1 inactivation [95].

### 2.2. Phosphatases are Downregulated at Mitotic Entry

Thousands of proteins are phosphorylated during mitosis by Cdk1, Plk, Aurora A and Aurora B, to name a few. They drive the dramatic cytological changes one associates with this phase: reorganization of cortical cytoskeleton to induce cell rounding and dissolution of the nuclear envelope in vertebrates, compaction of chromosomes to aid segregation and formation of a spindle structure to capture and biorient chromosomes at the metaphase plate. A coherent forward step towards this phase would dictate that opposing phosphatase activity be dampened, swinging the balance towards phosphorylation. Downregulation of many phosphatases occurs at this juncture through direct Cdk1-dependent phosphorylation or binding of various small, heat-stable inhibitors (see below).

Increasing Cdk1 kinase levels phosphorylate the highly conserved AGC kinase Greatwall. The two phosphorylated residues lie within the activation loop of the kinase domain and produce a partially activated enzyme. Intramolecular autophosphorylation of its C-terminal tail leads fully active Greatwall by stabilization of its active conformation [96,97].

Active Greatwall phosphorylates two small, heat-stable, intrinsically unstructured proteins: ENSA (α-endosulfine) and Arpp19 (cAMP-regulated phosphoprotein 19) [98,99]. Phosphorylation occurs at a Ser residue within a highly conserved motif present in both proteins and transforms them into potent inhibitors of PP2A-B55 holocomplexes. Specificity for the B55-bound form of PP2A arises as the proteins bind at the interface of B55 and the catalytic subunit, occluding the active site [100].

In keeping with a paradigm often governing enzyme-inhibitor interactions, PP2A-B55 itself is able to dephosphorylate p-ENSA/p-Arpp19, albeit very slowly, with a *k_cat_* two-three orders of magnitude lower than that of a generic PP2A-B55 substrate. PP2A-B55—p-ENSA *K_m_* is about four orders of magnitude lower than that for a phosphosubstrate, indicating very tight binding of the inhibitor. Strong inhibitor binding coupled with low rate of catalysis has been termed ‘inhibition by unfair competition’ [101]. This implies that as long as p-ENSA is replenished by active Greatwall, the mitotic state will be preserved through phosphatase inhibition. In summary, Cdk1 phosphorylation of Greatwall leads to its activation and phosphorylation of ENSA/Arpp19—inhibitors of PP2A-B55.

PP2A-B56 is present at kinetochores, where it phosphoregulates kinetochore-microtubule interactions, and is opposed by Aurora B. Phosphorylation of another small, heat-stable protein Bod1 recruited by Ndc80 causes it to inhibit PP2A-B56 complexes. This inhibition is proposed to maintain the correct balance of PP2A-B56 and Aurora B activities [102,103], although detailed mechanisms remain elusive.

Direct phosphorylation of PP2A subunits has also been reported to regulate enzyme activity by affecting assembly. Human PP2A catalytic subunit phosphorylation at its C-terminal tail at Thr307 prevented binding of B55 [104]. A recent study reported that phosphorylation of Thr174 of the budding yeast B55 by mitotic Cdk led to inhibition of its phosphatase activity [105]. Phosphorylation of the equivalent site Ser167 on human B55α, possibly by Cdk1-Cyclin B, inhibits holoenzyme assembly [106].

Cdk1 also directly phosphorylates PP1 near its C-terminus at Thr320 to inhibit its activity down to 30–40% of its peak levels [107,108,109]. Protein Kinase A phosphorylates Inhibitor-1 (l-1), a small heat-stable protein inhibitor, which then specifically inhibits PP1 [110]. In fact, inhibition by I-1 was often used to discriminate between PP1 and PP2A enzymes isolated from tissues in early studies [111]. Importantly, PP1 phosphorylation and I-1 inhibition are thought to act in concert to suppress its phosphatase activity. In an analogous paradigm to PP2A-B55 inhibition, PP1 itself removes these inhibitory phosphates, albeit slowly [110].

As previously mentioned, PP1 binding proteins often employ one or more short docking motifs to interact with the enzyme. Of its 200 known interactors, at least 90% employ the RVxF motif [11]. When the ‘x’ within the motif is a Ser or a Thr, it conforms to a basophilic motif preferred by the mitotic kinase Aurora B [112]. Indeed, Aurora B-mediated phosphorylation of these motifs prevents PP1 holoenzyme assembly during mitosis [113], representing another mode of PP1 inhibition.

PP6 is a phosphatase belonging to the PPP superfamily that also forms a heterotrimeric enzyme. It dephosphorylates the regulatory T-loop of Aurora A thus inhibiting its activity at centrosomes [114]. During mitosis, Cdk1 phosphorylation of the PP6 regulatory subunit creates a docking site for Plk1, which then phosphorylates the phosphatase complex at multiple sites and inhibits its activity through unknown mechanisms. Thus, PP6-mediated signaling is temporally inhibited to ensure a preponderance of Aurora A activity [115].

Another ubiquitous phosphatase of the PPP family, PP4, forms heterodimeric or heterotrimeric complexes in vivo [116,117], presumably leading to different substrate specificities or subcellular localization. For instance, *Drosophila* PP4 is targeted to centromeres via interaction of its regulatory subunit R3 with the centromere protein CENP-C, where it regulates centromeric integrity during mitosis [118]. It has also been implicated in mitotic centrosome function in *Drosophila* [119], *C. elegans* [120] and humans [121]. At the G2/M transition, rising Cdk1-Cyclin B activity leads to phosphorylation of a PP4 complex at centrosomes to inhibit its activity. As PP4 opposes γ-tubulin phosphorylation, its inhibition enables formation of the mitotic spindle [121].

During metaphase/anaphase, APC/C-mediated degradation of Cyclin B and various mitotic kinases sets in motion cascade of events that lead to reactivation of phosphatases (Figure 3). Thus, the APC/C is essential for phosphatase function during mitotic exit.

It is important to note that while there is a general swing towards kinase activation, some phosphatase activity is preserved throughout mitosis. Indeed, it is essential for mitotic progression. As is discussed in the next few sections, PP1 and PP2A-B56 activity at kinetochores plays an important role in stabilizing amphitelic kinetochore-microtubule linkages and silencing the SAC. These phosphatases have also been implicated in dephosphorylating the APC/C co-activator Cdc20 and initiating anaphase [122,123,124]. It is possible that association with certain structures within the cells precludes inhibitor binding, just as microtubule binding protects spindle assembly factors from APC/C-mediated turnover [125]. Another possibility is that these phosphatases are locally reactivated. How these complexes evade inhibition or achieve local reactivation is an important question for the future.

## 3. Building a Metaphase Cell

### 3.1. PP1 and PP2A Phosphatases Regulate Kinetochore Capture by Microtubules

Successful execution of chromosome segregation requires capture of kinetochore by microtubules emanating from spindle poles or centrosomes at the opposite ends of the cells. This ensures that each daughter cell gets one copy of the genome. Until all sister chromatids have correctly attached, kinetochores activate the SAC and hold cells in metaphase by inhibiting the APC/C. Thus, phosphatase mediated error correction and SAC inactivation are intimately linked to APC/C activation. In turn, APC/C degrades many proteins involved in these processes. Kinetochore capture is a stochastic process; many rounds of incorrect attachments precede mature, end-on attachments [126,127,128]. It is imperative that only bioriented chromosomes attached to the spindle be selectively stabilized.

How does the cell recognize correct attachments? On a molecular level, this occurs through warring activities of kinases and phosphatases at the centromere and kinetochore. Central to error correction is the kinase Aurora B, which is recruited to inner centromeres in two different ways. First, the mitotic Haspin kinase phosphorylates histone H3 at Thr3, which directly binds the Aurora B-containing Chromosome Passenger Complex (CPC) subunit Survivin [129]. Second, Bub1, a mitotic kinase, phosphorylates H2A at Thr120 at centromeres, which then leads to the recruitment of shugoshin-CPC [130]. Centromeric shugoshin contributes to another pool of Aurora B [131]. Upon autophosphorylation of its T-loop, possibly in trans, active Aurora B phosphorylates several kinetochore proteins and severs incorrect interactions with microtubules, thus giving them a chance to pursue correct ones. Among other proteins, the outer kinetochore subunit Ndc80 is the focal point of Aurora B kinase activity. Phosphorylation of its N-terminus reduces its affinity for microtubules and disfavors the connection [132]. Aurora B also phosphorylates the kinetochore localized Ska complex in metazoans, which is capable of efficiently binding curved ends of depolymerizing microtubule protofilaments. Thus, by helping kinetochores hold on to different microtubule architectures and states, the Ska complex enables ‘end tracking’. Phosphorylation reduces Ska-Ndc80 binding, dampening its affinity for the spindle microtubules [133,134]. Targets of Aurora B are also modulated by other kinases such as Cdk1, Plk1 and Mps1 in this endeavor [74].

A collaboration between PP1 and PP2A-B56 phosphatases, recruited through many mechanisms, leads to direct dephosphorylation of Aurora B substrates to strengthen kinetochore-microtubule interactions. PP2A-B56 directly binds the outer kinetochore, and serves to stabilize initial microtubule-kinetochore contacts, enabling them to form mature, end-on attachments [135]. B56-bound complex is drafted to an unattached outer kinetochore by directly binding the spindle checkpoint protein BubR1 via a LxxIxE motif. To give an example of the complex cross-talk at these regulatory foci, Cdk1 and Plk1 positively regulate PP2A-B56 binding in this context by phosphorylating residues within and downstream of the motif and enhancing B56 binding [24,25,26,27]. This population of the phosphatase opposes Aurora B phosphorylation of the PP1-binding RVxF motif in the outer kinetochore protein Knl1 and allows its recruitment [136]. PP1 can also be delivered to the site of action by binding to plus end-directed motors such as CENP-E. Analogous to its Knl1 binding, PP1 interaction with CENP-E is blocked by Aurora A- (at spindle poles) and B-mediated phosphorylation of a threonine within its RVxF motif. Once dephosphorylated, PP1-CENP-E stabilizes mature attachments [137]. Finally, the Ska complex can also directly engage PP1 [138].

As previously mentioned, Cyclin A is degraded during prometaphase by APC/C action, even though the spindle checkpoint is active. Timely degradation of Cyclin A at this juncture is essential for formation of stable kinetochore-microtubule (KT-MT) attachments [139]. The question remains of how Cyclin A evades the SAC. As kinase activity rises in mitosis, APC/C subunits are phosphorylated. The Cdk accessory subunit Cks1/2, in the form of Cyclin A-Cdk-Cks, independently recruits Cyclin A to the APC/C by binding phosphorylated residues within the latter and leads to its ubiquitylation [67,140]. Thus, APC/C action in prometaphase enables correct KT-MT attachments by limiting Cyclin A-associated kinase activity.

Once correctly attached, the two sister chromatids are pulled to opposite poles of the cell, thereby generating tension that is thought to place the kinetochore proteins outside the zone of influence of inner centromere localized Aurora B, more than 100 nm away [141]. Here, phosphatase activity predominates and preferentially stabilizes these attachments [142], through direct dephosphorylation of seminal targets such as Knl1 and Ndc80.

During mitotic exit, BubR1 (Mad3 in yeast, worms and plants) and Bub1 are targeted for degradation by the APC/C [143,144], shutting down PP2A-B56 recruitment and SAC signaling (discussed below). PP1, now in complex with Repo-Man, nips Aurora B signaling at the bud by dephosphorylating H3 p-Thr3 [145,146]. Aurora B is also physically evicted from the inner centromere to the central spindle. Dephosphorylation of budding yeast CPC subunit Sli15 (INCENP) by Cdc14 precipitates this event [147]. In humans, this role is performed by PP2A-B55 [21]. Finally, Aurora kinases are degraded by APC/C action in late mitosis, thus ensuring that their phosphosignaling is temporally constrained within the cell.

### 3.2. The Spindle Assembly Checkpoint Inhibits APC/C Activity in Metaphase and is Regulated by Phosphatases

Until all kinetochores are correctly captured and bioriented at the metaphases plate, the APC/C activity is held in check by a highly potent, diffusible complex that acts as a stoichiometric inhibitor—the Mitotic Checkpoint Complex (MCC). Even one unattached kinetochore can sequentially recruit an array of proteins to form the MCC, holding the entire cell in metaphase and safeguarding genome segregation.

The MCC is a complex composed of Mad2, BubR1, Cdc20 and Bub3. At unattached kinetochores, the Ndc80 complex, unfettered by microtubule binding, instead recruits the kinase Mps1 and drives a phospho-dependent signaling cascade. By virtue of being present at the outer kinetochore, Mps1 phosphorylates various proteins, including the MELT motifs of Knl1. This is the most upstream event in the MCC formation. As seen during error correction, Knl1 again acts as a scaffold and recruits Bub1-Bub3 complexes via its phosphorylated MELT motifs, which then recruit BubR1-Bub3 complexes. At this juncture, Mps1 directly phosphorylates Bub1 and leads to Mad1-Mad2 heterodimer binding [78]. Next, Mad1 phosphorylation by Mps1 stimulates its catalytic activity, and leads to conversion of open-Mad2 (o-Mad2), to the MCC-assembly competent closed-Mad2 (c-Mad2) by structural remodeling of the protein [148]. This process is critical to rapid MCC assembly and therefore instantaneous checkpoint response within the cell. Termed a ‘template-assisted mechanism’, Mad1-c-Mad2 heterodimers catalyze conversion of cytosolic o-Mad2 proteins to their closed conformers at unattached kinetochores. Importantly, spontaneous Mad2 conversion is disfavored outside the context of kinetochores as the protein faces a large energy barrier [149,150]. C-Mad2 is the structurally relevant form of the MCC. By capturing Cdc20 via its N-terminal KILR motif, it leads to MCC formation. MCC binds APC/C complexes already containing a bound Cdc20 co-activator and inhibits their activity [151]. Cdk1 and Plk1 kinases impinge at various stages throughout this process, for instance by directly phosphorylating Mps1 to enhance its kinase activity [74,152,153].

It was initially thought that the MCC functions by sequestering Cdc20 away from the APC/C. Recently, however, it has become clear that the MCC employs a multitude of mechanisms to target numerous aspects of APC/C function to obstruct its activity. Cryo-electron microscopy structures have revealed molecular details of APC/C-MCC interactions [61,64]. The hollow of the APC/C is occupied by the MCC complex and its own molecule of Cdc20. MCC binding causes the APC/C-bound Cdc20 to move away from Apc10, thus disrupting D-box binding site within the holocomplex. BubR1, by virtue of its various inhibitory degron motifs (D-box, KEN and the ABBA motifs), mounts a multi-pronged attack on the two Cdc20 molecules within the complex [56,61,64]. It caps all available degron-binding sites within Cdc20, hobbling any potential for substrate recruitment. In some conformations, BubR1 also precludes binding of the initiating E2 UbcH10 by occupying its binding site within the catalytic module at the base of the enzyme. In a different state however, possibly regulated by the Apc15 subunit of the enzyme, the MCC rotates away and vacates the initiator E2 binding site. How the APC/C switches between the two conformations is unclear. Subsequent UbcH10 binding to the second conformer allows ubiquitylation and degradation of the Cdc20 molecule within the MCC, thereby leading to MCC turnover [61,64]. Thus, APC/C-bound MCC is a dynamic, self-limiting inhibitor with an inbuilt ‘diffuse’ function. Cdc20 turnover is important for cells to recover from a SAC arrest and avoid cohesion fatigue. Cdc20 levels are replenished by synthesis during mitosis [154]; as long as kinetochores remain unattached, the MCC will continue to inhibit the E3 ligase. This process is continuously antagonized by the DUB Usp44 to maintain a fine balance of Cdc20 levels [155,156]. Another protein antagonizes MCC assembly: p31^comet^ adopts a Mad2-like fold, and extracts c-Mad2 from its complex with Mad1 and from within the MCC by directly dimerizing with it [157,158]. p31^comet^ also acts as an adaptor protein and delivers c-Mad2 to the AAA+ ATPase TRIP13, which then catalyzes its conversion to inactive o-Mad2 by ATP hydrolysis and inactivates the MCC [159]. Phosphorylation has been reported to regulate p31^comet^ activity in *Xenopus* and humans [160,161,162]. However, the details of the molecular mechanism are unclear, as is the nature of phosphatases that regulate this process.

Finally, in addition to MCC dissociation, further SAC signaling must also be extinguished at the source to ensure timely anaphase entry. Indeed, kinetochores proteins are in a state of flux due to presence of both kinases (Aurora B and Mps1) and recruited phosphatases (PP2A-B56-BubR1 and PP1-Knl1), which enables quick responsiveness of the system to changes in the input. Once the Ndc80 complex is bound to kinetochores, Mps1 can no longer bind and initiate its signaling cascade and MCC formation [163]. Direct dephosphorylation of Mps1 substrates is also required to prevent any potential for further MCC formation and is achieved by PP1 and PP2A phosphatases. PP1, recruited to outer kinetochores in a number of ways, is thought to take the lead in Mps1 substrate dephosphorylation [74]. PP1-Knl1 directly dephosphorylates Knl1-MELT motifs to halt any further binding of Bub1. PP2A-B56 bound to BubR1 is also thought to dephosphorylate Bub1, in addition to its role in recruiting PP1 by dephosphorylating its docking motifs within Knl1 [164,165,166]. As the same phosphatases regulate both error correction and SAC silencing, their interplay, in particular together with Aurora B and Mps1 kinases is complex. However, it is vital to understand how the antagonistic activities are integrated [74].

These molecular interactions underlie the highly responsive nature of MCC-mediated inhibition and explain how cells commence Cyclin B degradation as soon as the last chromosome is aligned [167].

### 3.3. Constructing and Segregating Mitotic Chromosomes

One of the hallmarks of the mitotic phase is the appearance of discrete, rod-shaped ‘condensed’ chromosomes, a prerequisite for accurate chromosome segregation to daughter cells. This is mediated by actions of two different SMC (structural maintenance of chromosome) protein complexes, condensin and cohesin [168,169]. They govern sister chromatid resolution, chromosome compaction and segregation, and are subject to regulation by kinases, phosphatases and the APC/C.

Condensin I and condensin II are ring-shaped multisubunit complexes that perform distinct functions in chromosome organisation and segregation in mitosis. In vitro reconstitution of mitotic chromatids has revealed that Cdk1 phosphorylation of condensin I is the sole mitosis-specific modification required for chromatid reconstitution [170]. Nonetheless, actions of Cdk1, Plk1 and Aurora B are also implicated in chromatin targeting of distinct condensin complexes and stimulating their activity [171,172,173,174,175,176]. In budding yeast, Cdc14 dephosphorylates the Smc4 subunit of condensin during mitotic exit [177]. In higher organisms, PP2A has been implicated in regulating condensin [178]. Additionally, PP6 has also been shown to positively regulate condensin I complexes by opposing inhibitory phosphorylation by casein kinase 2 [115,179].

Cohesin is a ring-shaped proteinaceous complex that holds together duplicated sister chromatids by physically entrapping them. Cell cycle-dependent acetylation of one of the cohesin subunits serves to ‘lock’ the ring in both yeast and vertebrates. Sororin, a vertebrate-specific protein is specifically recruited to acetylated cohesin. It protects cohesin from the ‘anti-establishment’ activity of a negative regulator, Wapl. Following entry into mitosis, vertebrate cells witness a removal of majority of cohesin complexes due to extensive phosphorylation of both Sororin and Wapl catalyzed by Cdk1, Plk1 and Aurora B. This occurs concomitant to condensin activity. Sororin phosphorylation destabilizes its interaction with cohesin, allowing Wapl activity to predominate [180,181]. Centromeric cohesin is preserved until anaphase through recruitment of PP2A-B56, which antagonizes these phosphorylation events and holds sister chromatids together until anaphases commences [182,183]. A mitosis-specific signaling cascade ensures the presence of PP2A-B56 complexes where their action is needed. As previously mentioned, Bub1 recruits Sgo1 by phosphorylating histone H2A at Thr120 [130]. Sgo1 directly binds PP2A-B56 through contacts with both the catalytic and regulatory subunits [184]. Bub1 degradation during anaphase turns off this pathway [185]; however, the identity of the phosphatase that resets H2A p-Thr120 is unknown.

Anaphase onset liberates separase by APC/C-Cdc20-mediated degradation of its inhibitor, securin. Centromeric cohesin is cleaved by this protease and sister chromatids commence their movement to opposite poles of the cell. A collaboration with PP2A-B56 phosphatase ensures that this step ensues in a switch-like fashion and precedes later events such as cytokinesis, which can damage any lagging chromosomes. CaMKII phosphorylates securin and makes it more amenable to APC/C-mediated degradation by increasing its degron-APC/C affinity. Separase-bound securin, however, is kept in a hypophosphorylated state by recruitment of PP2A-B56. Thus, free securin is targeted by the APC/C before separase-bound securin, ensuring that liberated separase is not re-inhibited by remaining securin molecules. Once activated, separase self-cleavage precludes PP2A binding and therefore re-inhibition by securin [186,187,188].

In budding yeast, securin degradation and decisive anaphase onset is also under phosphatase control, albeit with different underlying molecular details. Phosphorylation of yeast securin by Cdk1 impedes its degradation. As small levels of securin are degraded by the APC/C, Cdc14 is liberated, which dephosphorylates securin, promoting its degradation. Thus, a positive feedback loop is generated that leads to switch-like anaphase onset [189].

**Figure 4:**
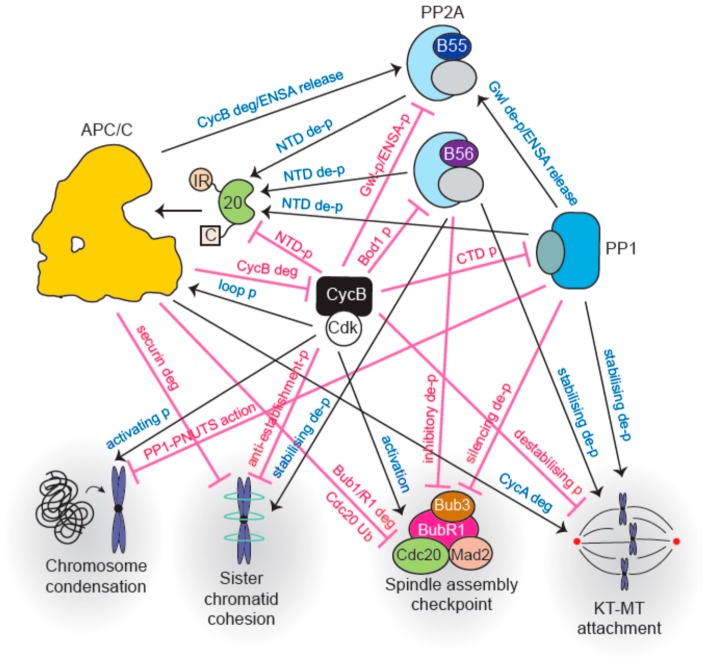
The APC/C and cell cycle phosphatases display complex interactions, influencing not only each other but also numerous events in mitosis and transition into anaphase. Black arrows indicate stimulation; red T-bars indicate inhibition. See text for details. Abbreviations: CycB, Cyclin B; de-p, dephosphorylation; deg, degradation; KT-MT, kinetochore-microtubule; p, phosphorylation; Ub, ubiquitylation.

Major interactions between cell cycle phosphatases and the APC/C are outlined in Figure 4.

## 4. Intricate Interplay between Phosphatases and the APC/C Regulates Metaphase-Anaphase Transition and Mitotic Exit

**Figure 5:**
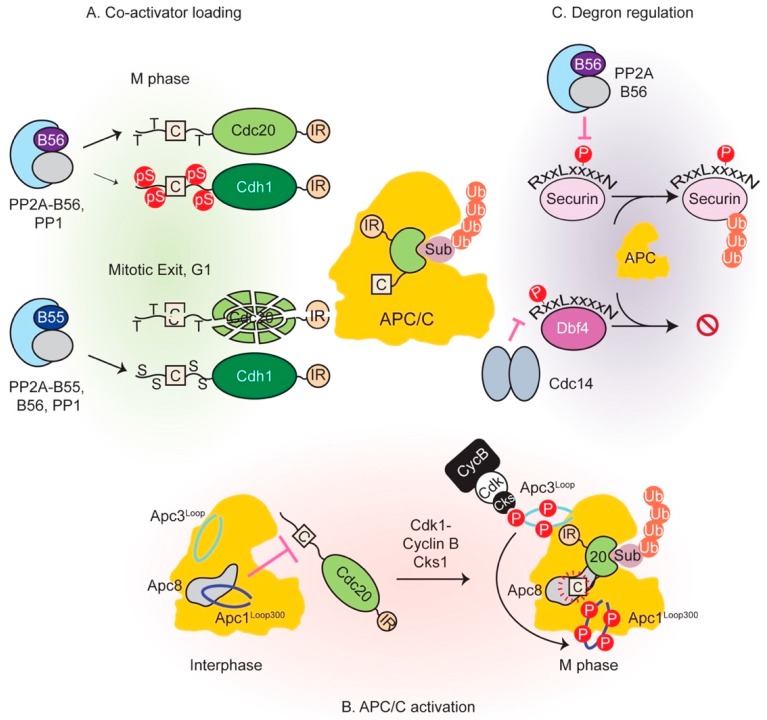
Phosphatases regulate multiple aspects of APC/C function. (**A**) In mitosis, PP2A-B56 and PP1 dephosphorylate phosphothreonine-rich Cdc20 N-terminus to promote its interaction with the APC/C. During mitotic exit and in G1, PP2A-B55 (and other mitotic exit phosphatases) dephosphorylate serine-rich Cdh1 N-terminus. In conjunction with other unknown mechanisms, this promotes co-activator exchange and Cdc20 degradation. (**B**) Apc1^Loop300^ occludes the C box binding site within Apc8 and prevents Cdc20 loading. Recruitment of mitotic kinases by Apc3^Loop^ leads to Apc1^Loop300^ phosphorylation (likely opposed by as-yet-unknown phosphatases) and Cdc20 loading. (**C**) Phosphoregulation of degrons influences substrate degradation by the APC/C. Securin phosphorylation at position 6 within the D-box stimulates its degradation. PP2A-B56 opposes this process. Budding yeast Dbf4 phosphorylation at position 2, removed by Cdc14, is refractory to its degradation. See text for details. Abbreviations: CycB, Cyclin B; Sub, substrate.

Once all chromosomes are aligned and the SAC is switched off, APC/C-Cdc20 becomes active. In addition to securin destruction, Cyclin B also gets degraded which reduces the overall kinase activity and initiates phosphatase reactivation. Extensive phosphoregulation of both the APC/C and co-activators governs its activity throughout mitosis. Phosphorylation precipitates opposite outcomes in this regard: phosphorylation of the APC/C activates it while co-activator phosphorylation prevents its association with the E3 ligase. Figure 5 summarizes control of APC/C function by phosphatases.

### 4.1. Phosphorylation of APC/C Enables Cdc20 Binding

Co-activators bind to APC/C at multiple sites, employing evolutionarily conserved regions within their largely intrinsically unstructured N- and C-terminal tails. These tails flank the highly structured WD40 propeller domain, which only mediates substrate recruitment rather than APC/C association. Of chief importance are two short linear binding motifs within co-activators (the C box and IR tail) that cooperatively enable Cdc20 and Cdh1 association with the APC/C by binding to the TPR lobe (the substrate recognition sub-complex). The C box, named so due to its evolutionary conservation within Cdc20 family of proteins [190], typically lies close to the N-terminus of co-activators and docks into one of the copies of the TPR subunit Apc8 [60,124]. The Ile-Arg or IR tail at the very C-terminus of co-activators binds an equivalent site within Apc3 [191]. The equivalence of the C box and IR tail receptor sites is demonstrated by the fact that the IR tail of the Cdc20 molecule within the MCC binds the Apc8 subunit instead [61,64]. Interestingly, the core subunit Apc10, the inhibitor Emi1, the E2 enzyme Ube2S and the prometaphase substrate Nek2A also employ the IR tail or variations thereof to associate with the APC/C [60,61,62,63,64,66].

Although both co-activators possess these binding sites, it had long been known that Cdc20 can only bind APC/C hyperphosphorylated in early mitosis by Cdk1 and Plk kinases [192,193,194,195,196]. Cdh1-mediated activation, however, is not conditional on APC/C phosphorylation status [193] and occurs towards the end of mitosis and G1. At this stage, APC/C phosphorylation is low due to Cdk1 inactivation by kinase degradation and Wee1 activity, and concomitant action of reactivated phosphatases. This dichotomy was resolved by cryo-EM, biochemical and functional analyses and unveiled a sequential phosphorylation relay that activates the APC/C by allowing Cdc20 binding [197,198,199]. Apc1 and Apc3 subunits were found to be hyperphosphorylated within two intrinsically unstructured loop domains: a large ~280 residue segment within Apc3, and a shorter ~100 residue stretch within Apc1 at position 300 from the N-terminus (Apc1^Loop300^), both of which are conserved in vertebrates. Mutation of Apc3 or Apc1 sites of phosphorylation to alanine considerably reduced APC/C-Cdc20 activity, with simultaneous mutation of both rendering it almost inactive and unable to bind Cdc20 [197]. It was found that Apc3 phosphomutant failed to recruit the phosphoadaptor subunit p9/Suc1/Cks [197], which had been known to interact as a part of cyclin B-Cdk1-Cks complex [200,201]. This led to downstream effects in the form of reduced Apc1 phosphorylation. Thus, initial phosphorylation of Apc3 by Cdk1 and polo-like kinases creates a phosphodocking site for p9/Suc1/Cks, probably through p-Thr residues that conform to its preferred docking motif [202,203], which then leads to intramolecular phosphorylation of Apc1^Loop300^. These results pointed to an inhibitory function for Apc1^Loop300^ that is relieved upon its phosphorylation. Cryo-EM studies revealed that this loop contained a stretch similar to the co-activator C box and physically occluded the C box binding site within Apc8 in the non-phosphorylated form of APC/C [198,199]. Phosphorylation of sites within Apc1^Loop300^ peels it away from this region by disrupting its interactions and allows Cdc20 binding. Consistent with this model, deletion of Apc1^Loop300^ in the context of the holocomplex permitted Cdc20-dependent activity even in the absence of phosphorylation [198,199]. Recombinant Apc1^Loop300^ could co-precipitate holo-APC/C while its phosphomimetic counterpart could not, further bolstering this conclusion [197]. Thus, intrinsically unstructured regions of the APC/C act as regulatory hubs to control its activity. It is conceivable that phosphatase recruitment to the APC/C is at least partly mediated through similar docking interactions. Recruited phosphatases could also be involved in dephosphorylating co-activator, thereby activating the complex (see below).

Phosphorylation only affects access of Cdc20 to the APC/C, with Cdh1 evidently outcompeting the autoinhibition of Apc1^Loop300^ to bind the enzyme. Higher affinity of Cdh1 compared to Cdc20 for the E3 ligase, evidenced by increased interaction sites within its N-terminus with multiple APC/C subunits, and reduced flexibility of the IR tail within Apc3 in cryo-EM structures has been suggested as an explanation for their differential behavior [198,204].

The budding and fission yeast APC/C adopts a very similar architecture to its vertebrate counterpart (barring the absence of Apc7) [205,206,207]. There is compelling evidence for phosphorylation-mediated activation of the yeast enzyme [192,208]. It is likely that this is general mechanism of cell cycle-mediated control of this E3 ligase and is evolutionarily conserved. A loop domain within Apc1, or within a different subunit in both yeasts might be implicated in this regulation.

A majority of APC/C subunits undergo phosphorylation [198,199]. Whether these impinge on the Apc3-Apc1 pathway or regulate other functions of the APC/C (such as MCC, Emi1 or phosphatase binding) remains to be elucidated.

At some point following entry into anaphase, APC/C dephosphorylation occurs and increased electrophoretic mobility of Apc3 is readily observed by SDS-PAGE [124]. Precisely when this occurs in the context of other cell cycle events is unknown, but it likely follows reactivation of PP1 and PP2A-B55 phosphatases. Dephosphorylated Apc3 would no longer support further Apc1 phosphorylation. Thus, it is possible that Apc1^Loop300^ is protected from, or impervious to dephosphorylation in early anaphase to preserve Cdc20 function. APC/C is not homogenously distributed throughout the cell. For instance, it is localized to mitotic chromosomes by the Ska complex in a dephosphorylated yet active form [209]. How this localization is mediated and how its hypophosphorylated state reconciles with our current view of APC/C function are unknown.

### 4.2. Co-Activator Dephosphorylation is Essential for APC/C Interaction

As mentioned previously, Cdc20 is an early activator of the APC/C, while Cdh1 takes the reins in late mitosis upon its dephosphorylation. The overall structure and organization of the co-activators is remarkably similar; nonetheless, important differences persist and define their characteristic behaviors. First, Cdc20 can only bind phosphorylated APC/C due to reasons outlined in the previous section. Secondly, both co-activators employ the use of D- and KEN boxes within their substrates; however, only vertebrate Cdc20 appears to bind the ABBA motif which contributes to its inhibition by BubR1 [56]. Budding yeast Cdh1 and Cdc20 also appear to have slight variations within their preferred ABBA motif signatures [58,210]. Finally, in the absence of Cdh1, Cdc20 is able to compensate for most of its functions, possibly due to its stabilization as it is degraded by action of APC/C-Cdh1. Efficient Aurora A and B degradation, however, appears to be dependent on Cdh1 [211].

In contrast to APC/C, Cdk1 phosphorylation of both Cdc20 and Cdh1 is inhibitory and prevents their association with the E3 ligase [124,212,213,214]. In early anaphase, Cdc20 phosphorylation at several sites within its N-terminal tail reduces its affinity for the APC/C in an additive manner. Preserving the function of any one site while mutating the rest does not elicit the same inhibitory effect. Nonetheless, a p-Thr site flanking the Cdc20 C box appeared to be particularly inhibitory and phosphorylation of three threonine residues (Thr64/Thr68/Thr79 in Xenopus Cdc20) largely inhibits the activation role of Cdc20 [124]. Coupled with the observation that the C box flanking phosphosites are disordered within cryo-EM structures [198,199], it appears that negative charges and/or bulky phosphate groups destabilize C box receptor interactions [197].

As Cyclin B is degraded, the previously inhibited phosphatases PP1 and PP2A-B55 get reactivated (discussed later). Cdh1 is dephosphorylated, probably primarily by active PP2A-B55 in vertebrates [21], and replaces Cdc20 as the co-activator. Prior to late anaphase, phosphorylation of Cdh1 at its N-terminus precludes its APC/C binding [193,212]. Analogous to Cdc20 regulation, Cdh1 phosphorylation destabilizes the interaction of its C box and other affinity elements with the APC/C [60].

A recent study addressed how PP2A-B55 creates distinct temporal windows for co-activator dephosphorylation and activation: while Cdc20 is dephosphorylated in early anaphase, Cdh1 is only targeted later. Phosphothreonine residues within Cdc20 are preferred PP2A-B55 substrates compared to phosphoserines, a property likely shared by all members of the PPP family owing to the similarities of their catalytic cores. This allows faster activation of Cdc20 by preferential dephosphorylation of its residues that are mostly Thr. In contrast, p-Ser residues predominate Cdh1 N-terminus and are disfavored PP2A substrates. Their dephosphorylation occurs later in anaphase when other factors such as reduced substrate competition possibly make its dephosphorylation more amenable. Importance of late Cdh1 dephosphorylation is underscored by the aberrant mitotic phenotypes associated with a phosphothreonine variant of the co-activator that is dephosphorylated earlier [21]. How PP2A enzymes select a p-Thr is unknown. It has been suggested that a small hydrophobic cleft close to the metal ion binding sites at the catalytic core could make additional contacts with the extra methyl group that differentiates a threonine from a serine [215]. This could translate into p-Thr residues exhibiting a lower *K_m_*, a higher *k_cat_* or both, instigating faster turnover. In summary, both Cdc20 and Cdh1 are dephosphorylated at different points in the cell cycle to drive APC/C activation.

Cdc20 dephosphorylation invokes a catch-22 scenario: on the one hand it requires PP2A-B55 activity; on the other, it promotes B55 reactivation by degrading Cyclin B [216]. It begs the question: how does initial Cdc20 activation take place? Could a very small pool of B55 have escaped p-ENSA inhibition at metaphase to promote Cdc20 dephosphorylation? While this possibility cannot be ruled out, given the stoichiometric excess of p-ENSA over PP2A-B55 and subnanomolar *K_d_* [101], it is likely that initial Cdc20 dephosphorylation is commenced by other phosphatases. Kinetochore-localized PP1-Knl1 was shown to opportunistically dephosphorylate Cdc20 recruited by the ABBA motif within Bub1 in *C. elegans* [122]. However, it is likely that other sources of phosphatase activity also target Cdc20, as abrogation of the PP1 pathway leads to mitotic delay rather than a block. PP2A-B56 represents another possibility as it is active in mitosis. In fact, PP2A-B56 has been shown to associate with the APC/C constitutively [123], although the molecular basis of this interaction remains unknown. It has also been reported that PP2A-B56 promotes the stable association of Ube2S with the APC/C through dephosphorylation of Cdc20 at Ser92, although the detailed mechanism remains elusive [217].

### 4.3. Multiple Mechanisms Regulate Degron-Mediated Substrate Degradation

Mitotic exit witnesses degradation of at least a 100 protein substrates in a strict temporal order—a prerequisite for successful division. Perturbations in this order upset cell division. Coupled with the fact that protein synthesis exacts a considerable energetic cost, cells have evolved mechanisms to ensure specific, timely and efficient degradation of substrates by the APC/C. Computational modeling of APC/C-Cdc20-mediated substrate degradation in yeast concluded that substrate affinity, catalytic rate of ubiquitin transfer to achieve multi-ubiquitylation and substrate competition can together determine the order of substrate degradation [218]. Co-activator WD40 domains recruit substrates by employing structured groves on their surface that bind degron motifs. Degron requirement can be bypassed by independently recruiting substrates to the E3 ligase by a translational fusion of a substrate-APC/C subunit for instance [52]. Structural and functional analyses have revealed the three major degron-binding pockets: D-box (RxxLxxxxN) binds between a conserved region between WD40 blades 1 and 7, while residues C-terminal to it contact the Apc10 co-receptor; KEN box (composed of residues Lys-Glu-Asn) binding is mediated by the top of the WD40 barrel; and the ABBA motif (Fx[ILV][FY]x[DE]) receptor is situated within a hydrophobic surface formed between blades 2 and 3 [219,220,221].

There is considerable plasticity among what constitutes a ‘degron’ motif and seemingly incompatible residues can be allowed in a particular sequence context. For instance, divergence from consensus can be tolerated if contacts at additional positions stabilize binding, a concept reviewed by Davey and Morgan [58]. The degeneracy of the degron binding pocket permits multiple flavors of degrons to bind likely with a variety of affinities, contributing to their orderly degradation.

Multivalent substrate interactions, either with the WD40 domains by presence of multiple degron motifs, or with other APC/C subunits can elicit higher affinity—a strategy often employed by APC/C inhibitors. Higher order properties can also determine substrate binding and degradation. The example of Nek2A degradation illustrates this concept: the MR tail of Nek2A resembles co-activator and Apc10 IR tails and allows it to be independently bound and degraded by the APC/C during prometaphase, when the SAC is active. Although necessary, the MR tail is not sufficient for degradation; dimerization of Nek2A is also essential [222]. It is likely that dimerization works by increasing affinity of Nek2A for the APC/C, as docking of one molecule by the MR tail would obligatorily bring in a second.

Degron modification is another major mechanism regulating substrate degradation. Acetylation of BubR1 during metaphase blocks its degradation during metaphase [143]. Additionally, phosphorylation within and around degron motifs can occasion substrate degradation or stabilization and is highly context specific. For instance, budding yeast securin phosphorylation on Cdk1 residues situated outside of the D- and KEN box motifs is refractory to degradation. Activation of the Cdk1-antagonizing phosphatase Cdc14 by separase leads to securin dephosphorylation and degradation in a positive feedback loop (as mentioned in Section 3.3) [189]. On the other hand, phosphorylation of human securin Thr66 that lies within the core of the D-box motif enhances its interaction with the co-activator and promotes degradation [186,219]. This forms the molecular basis of separase stabilization by its previously mentioned PP2A-B56 association [186]. Further highlighting the importance of the precise position of phosphoresidues in relation to degron motifs are the following examples of Dbf4 and Cdc6. Phosphorylation of budding yeast DDK kinase subunit Dbf4 one residue after the Arg within its N-terminal D-box delays its degradation in vivo, indicating that phosphorylation at this position is inhibitory [210]. Cyclin E-Cdk2 phosphorylates human Cdc6 at two Ser sites, each N-terminal to a D- and a KEN box and reduces its association with Cdh1 in G1. This blocks Cdc6 degradation to allow timely origin licensing and S phase initiation [223].

## 5. Phosphatases Escort Cells through Exit from Mitosis

Phosphostate of a protein is always in flux; it is a readout of the equilibrium kinase and phosphatase activities governing it. Any change in one of the parameters alters this equilibrium and is swiftly reflected in the phosphate occupancy of the protein. This is the principle behind rapid phosphatase activation as cells commence exit from mitosis. Cyclin B degradation by APC/C-Cdc20 decreases Cdk1 activity and creates a domino effect. The balance is shifted towards PP1 autoactivation by dephosphorylation of its C-terminal tail and I-1 [108,109,110]. Free PP1 then dephosphorylates and partially inactivates Greatwall [224,225,226], ceasing the supply of phosphorylated ENSA/Arpp19. Any PP2A-B55 dephosphorylated p-ENSA/p-Arpp19 inhibitor molecules are no longer replenished. Williams et al. estimated that these inhibitors are present in a 5-10 fold excess to PP2A-B55, and would be dephosphorylated within a few minutes of Greatwall inactivation to swiftly reactivate the phosphatase [101]. PP2A-B55 completes Greatwall inactivation by full dephosphorylation of the protein. This positive feedback loop, coupled with the rest of the system architecture makes mitotic exit a bistable transition [226]. Once liberated, PP2A-B55 is thought to dephosphorylate hundreds of substrates in conjunction with PP1 and PP2A-B56. In fission yeast, a similar principle of phosphatase activation governs, although the molecular underpinnings are unclear. PP1 liberated upon Cyclin B degradation activates PP2A-B55 by directly binding it. Active PP2A-B55 then dephosphorylates a PP1 docking site within PP2A-B56, thereby allowing PP1 to bind and activate it [227]. PP1 could act by reversing inhibitory B55 phosphorylation mentioned previously [105,106]. How it effects B55- and B56-bound PP2A enzymes is a question that remains to be answered.

This leads us to an important discussion point: there likely is not one ‘mitotic exit phosphatase’ in higher organisms. In contrast, inactivation of Cdc14 in budding yeast elicits a complete block and arrests cells in telophase with elongated spindles due to its role in downregulating kinase activity [213,228,229]. Mitotic exit in human cells and other vertebrates such as *Xenopus* has only been prevented upon pan-phosphatase inhibition by toxins such as okadaic acid (OA) (following Cdk1 inhibition, for instance) [19,230,231,232], which inhibits multiple phosphatases to varying degrees. Nonetheless, that phosphatases collaborate to bring about robust mitotic exit in all organisms is incontrovertible.

Assembly of a central spindle, nuclear envelope reformation, chromosome decondensation and finally abscission into two daughter cells along the cytokinetic furrow are some of the events associated with mitotic exit. PP2A-B55 directly dephosphorylates p-Thr481 within Prc1, allowing its association with the central spindle where it bundles microtubules in an antiparallel array and defines the position of the cytokinetic furrow. PP2A-B55 also controls dephosphorylation of Aurora A regulator Tpx2 and its central spindle localization [20]. PP2A-B56 recruited by Kif4a also influences central spindle growth by opposing Aurora B phosphorylation of Mklp2 and Kif4a [21,233]. PP2A-B56 is also recruited by the centralspindlin protein Racgap1 and regulates its phosphorylation for accurate execution of cytokinesis. As mentioned previously, APC/C-Cdh1 activity later degrades Aurora B and halts further signaling.

PP1 also plays a major role in regulating mitotic exit and cytokinesis [234,235]. For instance, PP1 in conjunction with its regulatory subunit Repo-Man dephosphorylates H3 p-Thr3 to evict Aurora B from centromeres to the central spindle. Repo-Man association with histones is held in check by inhibitory phosphorylation by Cdk1. Cyclin B degradation changes the balance of kinase/phosphatase activities and allows PP1 and PP2A-B56-mediated (via a LxxIxE motif) dephosphorylation of Repo-Man, efficient PP1 binding and centromere targeting. PP1-Repo-Man has also been implicated in reestablishment of the nuclear envelope [145,146,236] and dephosphorylation of importin β to initiate nuclear envelope reassembly [236]. PNUTS (PP1 Nuclear Targeting Subunit), another PP1 interacting protein binds chromatin, and has been shown to enhance in vitro chromosome decondensation [237].

This is far from an exhaustive list of phosphatase regulation of mitosis; one can nonetheless appreciate that it is essential that these events occur in a specific order to avoid mitotic failure. For instance, premature recruitment of Prc1 to the central spindle upon Greatwall depletion induces early cytokinesis and causes a ‘cut’ phenotype in a majority of cells [232]. Therefore, not only must phosphatases be activated at the right time, they must also dephosphorylate hundreds of substrates in the right order. In *Xenopus* egg extracts and human cells, high levels of non-degradable Cyclin B block cells in metaphase, intermediate levels allow progression into a pseudo-anaphase state with separated sister chromatids, while persistent low levels arrest cells in telophase [238,239]. These observations suggest that ordering mechanisms exit in higher vertebrates. Differences in PP2A-B55 activation as a function of Cyclin B levels can partly explain some of these observations [232]. However, once liberated PP2A-B55 must still contend with a multitude of substrates in the backdrop of opposing activities of mitotic kinases such as Plk1. In budding yeast, the relative catalytic efficiencies of Cdk1 kinase and Cdc14 phosphatase towards a particular substrate was shown to set its dephosphorylation time and order mitotic exit [240]. It is likely that similar mechanisms exist in higher organisms, albeit with different molecular players.

How are hundreds of proteins discriminated by mitotic phosphatases? Part of the answer lies in intrinsic preference for phosphothreonines exhibited by PP2A phosphatases. Accordingly, survey of proteins dephosphorylated very early during an induced mitotic exit in human cells revealed that p-Thr-Pro (p-TP) sites were significantly more dephosphorylated than p-Ser-Pro (p-SP) sites by OA sensitive phosphatases [19]. A small non-polar residue at the +2 position potentiated, while a +2 Pro made p-TP refractory to dephosphorylation [19]. In another study of human cells undergoing mitotic exit as a result of phosphatase activation upon mitotic extract dilution, 45% of candidate PP2A-B55 dependent substrates were p-Thr, compared to 25% p-Thr detected in the total phosphoproteome [20], corroborating previous observations. Patches of basic residues both N- and C-terminal to the phosphosite enhanced dephosphorylation and depended on an acidic surface within B55. In fact, a correlation between motif basophilicity and PP2A-B55 was uncovered [20]. Highly basophilic motifs would be disfavored by Plk1, perhaps further contributing to their enhanced dephosphorylation [241]. A phosphoproteomic study uncovered that PP6, a phosphatase highly similar to PP2A, instead prefers acidophilic motifs within substrate proteins and opposes CK2 phosphorylation [242].

A majority of cellular phosphorylation events are catalyzed on serine residues (98.2%) [243]. This could be due to their greater overall numbers in the proteome and higher surface accessibility compared to the slightly more hydrophobic nature of threonines. How is their dephosphorylation controlled? Presence of bulky aromatics upstream to the p-Ser enhances their dephosphorylation by PP2A-B55 and constitutes one mechanism, although its molecular basis is unknown [20]. It is also possible that, on an average, p-Ser are targeted later in mitosis when p-Thr residues no longer outcompete them, as substrate competition has been shown to be an ordering mechanism [240]. Finally, it is also likely that a serine-specific phosphatase collaborates with PP2A enzymes. Cdc14 is a p-SPxK/R-directed phosphatase [244,245] and could aid dephosphorylation of some substrates, particularly in early anaphase. Indeed, human Cdc14B has been shown to catalyze phosphoserine-rich Cdh1 dephosphorylation upon DNA damage in G2 to induce Plk1 degradation and halt the cell cycle [246]. Whether a supportive role for Cdc14 in normal mitotic dephosphorylation also exists remains a question for the future.

Finally, most proteins contain multiple phosphosites that could be potential phosphatase targets upon binding to substrate docking motifs such as LxxIxE. How the presence of these substrate affinity elements colludes with phosphosite preferences in the context of the whole protein is unknown.

Our discussion thus far has been simplified by referring to each phosphatase as a homogeneous entity. This view, however, does the complexity of the system a disservice: each phosphatase has multiple isoforms. Do they exist to provide redundancy and buffer the system against inactivating mutations? Or do they perform distinct functions within the cell? A recent report examined the importance of multiple B56 isoforms, all of which contain an LxxIxE-binding pocket but exhibit distinct localizations within cells: while B56α binds Sgo2 at centromeres, B56γ prefers BubR1 binding via its LxxIxE motif. A small loop within B56α is thought to prevent docking interactions with BubR1 by repressing its binding to LxxIxE motifs, and enabling Sgo2 interactions instead [247]. Specific binding of PP1γ to its regulatory proteins Repo-Man and Ki-67 follows a similar theme: presence of an N-terminal Arg residue generates a regulator binding pocket unique to PP1γ [248]. Therefore, there has been considerable subfunctionalization between different phosphatase isoforms, providing finer brushstrokes on the canvas of mitosis. Which substrates are targeted by different phosphatase pools remains unknown. Coupled with structural basis for differential substrate targeting, this knowledge can aid development of drugs specific to a particular flavor of phosphatase.

## 6. Roles in Diseases and Avenues for Therapies

As the APC/C and phosphatases have pivotal roles in the cell cycle and beyond, they have been implicated in various diseases such as cancer. In case of the APC/C, heterozygous mutations within core subunits Apc3, Apc8 and Apc6 are found in human colon cancer cells [249]. Involvement of the APC/C co-activators has also been found in tumorigenesis and disease progression. Overexpression of Cdc20 in particular has been correlated to poor prognosis in various cancers such as non-small cell lung cancer [250], colorectal cancer [251], multiple myeloma [252], etc. Studies in cancer cell lines showed that depletion of Cdc20 caused cell cycle arrest and a reduction in cell viability, underlining active participation of Cdc20 in disease state in addition to its value as a prognostic marker. Further bolstering the role of Cdc20 as a potential oncogene are observations in mouse skin cancer models, where inducible depletion of Cdc20 led to regression of tumors due to cell cycle arrest followed by apoptosis [253]. Cdh1 is thought of as a tumor suppressor, perhaps due to its role in restraining the entry of cells into the cell cycle and maintaining genome stability. Accordingly, several cancers exhibit a reduction in Cdh1 levels or function [254,255,256], due to hyperphosphorylation [257], for instance. Cdh1 heterozygous deletion mice are more susceptible to developing tumors later in life [258]. Furthermore, Cdh1 also regulates myriad aspects of development and functioning of the nervous system [259], and its downregulation has been implicated in Alzheimer’s disease [259,260,261].

APC/C has been proposed as a potential target in cancer therapy, as its inhibition leads to an arrest in mitosis followed by cell death [262]. Two small molecule inhibitors of the APC/C, TAME (tosyl-l-arginine methyl ester) and apicin were isolated from screens in *Xenopus* extracts [263]. TAME blocks the structurally equivalent C-box and IR binding sites within the APC/C and prevents efficient co-activator loading [198]. Apicin occludes the D-box receptor within Cdc20 and competes with APC/C substrates. Simultaneous inhibition of the APC/C with these two inhibitors has a synergistic effect in blocking mitotic exit [264]—a strategy in clinical use with SAC inhibitors. Indeed, SAC inhibition alone appears to be inefficient in blocking mitotic exit, as cells escape the arrest due to leaky Cyclin B degradation [265]. Combination therapy with APC/C inhibition is likely to constitute a more effective anti-cancer strategy. Additionally, due to the tumor suppressive activity of APC/C-Cdh1, it is desirable to specifically target APC/C-Cdc20 complexes. It is thus important to understand the differences between loading of these co-activators.

PP2A has been heavily implicated in several diseases such as cancer, Alzheimer’s disease, diabetes, etc. [266,267]. Traditionally, PP2A has been thought of as a tumor suppressor, with its downregulation promoting cancer development. For instance, PP2A subunits have been frequently found to be mutated in cancer. Cancer-associated mutations within the scaffolding [268,269], catalytic [270] and regulatory subunits [271] typically tend to affect assembly of the holoenzyme. Endogenous inhibitors of PP2A such as SET have also been found to be upregulated in cancer cells [272,273], thereby leading to a reduction in PP2A activity. Following these observations, activation of PP2A has been suggested as a potential cancer therapy, by inhibiting SET [274], or in combination therapy to prevent PP2A inhibition-mediated drug resistance [275]. Conversely, PP2A inhibition has been employed to overcome resistance of some cancers to radiation therapy [276]. To this end, a small molecule PP2A inhibitor called LB-100 has entered clinical trials for treatment of solid cancers among others [277]. However, LB-100 also exhibits inhibition of PP5 [278], owing to the previously mentioned similarities in the catalytic sites of PPP family of phosphatases. Therefore, it is vital to further understand the differences between phosphatases relevant to diseases to develop specific inhibitors. Targeting PP1 has also been proposed as an anti-cancer therapy as its inhibition in mouse xenograft models impaired tumor growth [279]. As PP1 binds to hundreds of regulatory proteins, its blanket inhibition is likely to be highly toxic. Recent drug screening approaches have sought to target specific PP1-regulator holocomplexes in treatment of Huntington’s disease [280], for instance.

Cell cycle phosphatases have been implicated in numerous signaling pathways, regulating myriad functions. A systems-level analysis of the consequences of phosphatase perturbation in various contexts would be a beneficial complement to various studies.

## 7. Outlook

Mitosis is a tour de force achieved by phosphatases and the APC/C working in concert with mitotic kinases. They integrate a variety of information to ensure timely and accurate execution of cell division. Inhibitors, regulatory modifications and opposing activities modulate their activities to fine-tune passage through cell cycle transitions. The past few years have unfurled layers of regulation by critical molecular pathways that control their activities.

A larger theme emerging in phosphatase and APC/C function is the ubiquity of regulation by intrinsically unstructured loop domains. These act as regulatory hubs to occasion myriad effects: they underlie efficient catalysis by increasing substrate residence time or local concentrations of regulators with respect to the enzyme. The example of PP1 shows that one enzyme can possess multiple pockets that bind their own short linear motifs. Thus, it is likely that many other such interactions within other phosphatase and the APC/C await discovery.

Equilibrium protein modification relies on many factors. Some of them are intrinsic to the substrate, such as the nature of the phosphosite presented to phosphatases, or indeed the accessibility of the substrate lysines by the APC/C. These can be further potentiated by the strength of degrons, or presence of docking motif interactions. However, many factors extrinsic to the substrates are likely to exert equally important effects on protein modifications. Higher local concentrations of enzymes, achieved by tethering them to cellular structures for instance, would aid the formation of substrate-enzyme complex and catalysis. Competition provided by more efficient substrates could also delay enzyme action on less preferred ones, as would the presence of opposing enzyme activities. Hence, unraveling how these factors work together would help us better understand how two cells are successfully produced from one.
